# The COVID-19 Pandemic and Emergencies in Otolaryngology–Head and Neck Surgery: An Analysis of Patients Presenting to Emergency Rooms in South-West Germany: A Bi-Center Study

**DOI:** 10.3390/diseases12080194

**Published:** 2024-08-22

**Authors:** Stephan Wolpert, Nora Knoblich, Martin Holderried, Sven Becker, Thore Schade-Mann

**Affiliations:** 1 Department of Otorhinolaryngology, Head & Neck Surgery, University Hospital Tübingen, BW, Elfriede-Aulhorn-Straße 5, 72076 Tübingen, Germany; stephan.wolpert@med.uni-tuebingen.de (S.W.);; 2 Department of Medical Development Process and Quality Management, University Hospital Tübingen, 72076 Tübingen, Germany; 3 Institute of Health Care and Public Management, University of Hohenheim, 70599 Stuttgart, Germany

**Keywords:** COVID-19, emergency room, otolaryngology–head & neck surgery, overcrowding

## Abstract

This study was designed to examine the changes in emergency room visits in otolaryngology, head and neck surgery, during the COVID-19 pandemic. The study included 11,277 patients who presented to a tertiary care hospital (ER) and an emergency practice (EP) during on-call hours in the first half of 2018, 2019, and 2020. The epidemiologic parameters, diagnoses, and level of urgency were recorded using a four-step scale. A comparison was made between the pre-pandemic years and 2020. The findings revealed a significant decrease in the frequency of ER visits in the second quarter of 2020 compared to 2019 (ER: 30.8%, EP: 37.8%), mainly due to the fact that there were significantly fewer patients, with low levels of urgency. Certain diagnoses, such as epistaxis (−3.0%) and globus sensation (−3.2%), were made at similar frequencies to 2019, while inflammatory diseases like skin infections (−51.2%), tonsillitis (−55.6%), sinusitis (−59%), and otitis media (−70.4%) showed a significant reduction. The study concludes that patients with a low triage level were less likely to visit the ER during the early stages of the pandemic, but some diagnoses were still observed at comparable rates. This suggests a disparity in perception between patients and ER staff regarding urgency. Many of the issues discussed were also emphasized in the 2024 proposal by the German Ministry of Health to reform emergency care in Germany.

## 1. Introduction

Emergency department overcrowding is a longstanding key issue for timely and appropriate emergency care worldwide [1,2]. During the global coronavirus disease (COVID-19) pandemic, significant changes in healthcare delivery profoundly impacted emergency care. COVID-19, caused by the severe acute respiratory syndrome coronavirus 2 (SARS-CoV-2), first emerged in China in late 2019 [3]. The virus subsequently spread globally, initially causing localized epidemics and eventually leading to a worldwide pandemic [4]. In March 2020, Germany experienced an exponential increase in COVID-19 cases, resulting in a dramatic surge in intensive care patients and raising concerns about the potential collapse of local healthcare systems. Consequently, elective medical procedures and surgeries were postponed [5,6]. This unprecedented disruption of healthcare services heightened awareness of the looming healthcare crisis, probably influencing the utilization of emergency care facilities. At German otorhinolaryngology, head and neck surgery (OHNS) university centers, approximately 35,000 outpatient appointments and around 12,000 surgical interventions were postponed between 15 March and 15 April 2020 [5]. In our center, we prioritized emergency surgeries for acute life-threatening conditions and urgent elective surgeries, such as oncology cases.

The Robert Koch Institute (RKI), Germany’s Federal Agency for Disease Control and Prevention, routinely provides weekly reports on emergency department visits from eight representative departments across the states of Baden-Württemberg, Bayern, Niedersachsen, Sachsen, and Schleswig-Holstein using Surveillance Monitor (SUMO) system which was established in 2020 by the RKI [7]. This software was developed to process patient data for surveillance and public health research [7]. Surveillance data indicated a reduction in emergency department usage during the first wave of the COVID-19 pandemic (March to June 2020). Additionally, a study analyzing general healthcare emergency department visits in Germany during this period yielded similar findings [8]. Notably, there was a recorded decrease in certain life-threatening diagnoses, such as acute myocardial infarction and stroke [8]. However, a detailed breakdown of the diagnoses and the urgency of the visits was not provided.

Several publications document reductions in specialized emergency department visits during the pandemic, indicating shifts in patient behavior and healthcare utilization [8,9,10,11]. However, existing research often has limitations, such as a focus on single centers, a lack of detailed triage systems to adequately assess the urgency of visits, and limited temporal analysis. The concentration on single-center data restricts the generalizability of the findings. Similarly, studies emphasizing the pandemic’s impact on OHNS services frequently lack specialized triage systems. Additionally, some research only examines patient flow in regions with low COVID-19 prevalence [12]. These studies collectively highlight a critical gap in the literature: the need for comprehensive, multi-center analyses that include detailed diagnostic categorization and specialized triage systems tailored to OHNS emergencies.

This study was designed to understand the changes in consultations with a specialized otolaryngology, head and neck surgery emergency room (ER) and to close the gap in the literature detailed above.

## 2. Materials and Methods

We focused our analysis on the first wave of cases in Germany to exploit the novelty and unprecedented nature of the SARS-CoV-2 pandemic and its unique implications for emergency healthcare. Therefore, we designed a retrospective analysis of patients presenting to the ER of the Department of OHNS at the University Hospital of Tübingen (OHNS ER, referred to as “ER”) during the first surge of COVID-19 cases in Germany and the county of Tübingen, which occurred during the first two quarters of 2020. The setting of our ER is a specialized OHNS ER at a university hospital that serves a tertiary referral center and local provider at the same time. The inclusion criterion was presentation without an appointment during on-call hours. ER consultations from other internal departments and scheduled patient referrals from other hospitals were excluded. The on-call hours for the ER are Monday–Thursday from 5 pm to 7 am, Fridays from 1 pm to 9 am, Saturdays and public holidays before weekends from 8 pm to 9 am, and Sundays and public holidays before business days from 8 pm to 7 am. The medical record system was screened for patients fulfilling the inclusion and exclusion criteria and presenting within the eligible time frame.

**Figure 1:**
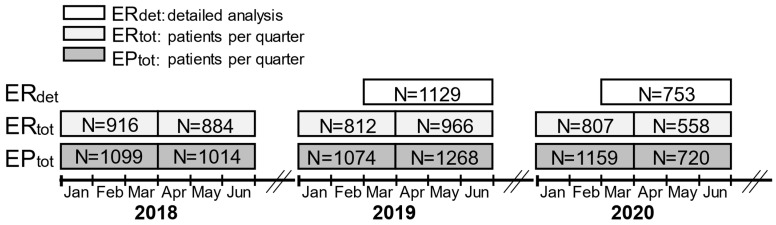
We analyzed two different cohorts: (1) The ER and (2) the EP. For the years of 2018 to 2020, the absolute number of consultations was recorded (ERtot and EPtot). For the ER, we had access to a detailed database (ERdet). The numbers in the boxes represent the included consultations. The months are abbreviated: Jan: January; Feb: February; Mar: March; Apr: April; May: May; Jun: June.

To facilitate an annual comparison and account for seasonal fluctuations (e.g., respiratory infection other than SARS-CoV-2) the two pre-pandemic years of 2018 and 2019 were included in the retrospective analysis. The total number of daily consultations for the years 2018 to 2020 from January 1 to June 30 was recorded (Figure 1). For the period from March to the end of June 2019 (25 February 2019–30 June 2019) and 2020 (24 February 2020–5 July 2020), additional features including sex, date of birth, diagnosis, reason for presentation (e.g., surgical complications and place of initial surgery), and type of treatment (outpatient, hospitalization, surgery) were recorded. To facilitate weekly analysis, the time frame was adjusted to include complete weeks.

The ER as a tertiary referral center might be biased in terms of consultation frequency and case severity. To account for this, the “Tübingen ear, nose and throat emergency practice” (ENT EP, referred to as “EP”), a more generalized center, was included. The EP primarily serves outpatients on weekends (Saturdays and Sundays) and national holidays from 8 am to 8 pm and is run by physicians in private practice. The same inclusion and exclusion criteria were used. Data for the EP were provided by the Association of Statutory Health Insurance Physicians (“Kassenärztliche Vereinigung”). Due to the retrospective nature and deviation documentation standards, only the numerical data of visits without details regarding diagnosis and urgency were available.

In summary, this retrospective study analyzes data from two distinct patient cohorts: (i) patients presenting to the ER and (ii) patients presenting to the “Tübingen ear, nose and throat emergency practice” (ENT EP, referred to as “EP”). Including both centers, located in the city of Tübingen, provided a more representative view of OHNS emergencies, as the patient demographics of a university hospital (a tertiary center) may differ from those of an emergency practice.

Figure 1 offers a visual overview of the different cohorts and timeframes.

To address the lack of an urgency-based characterization, we categorized patients based on the urgency of their symptoms and diagnoses (Table 1). To assess the urgency (also as a surrogate for the appropriateness of presentations in our ER), we developed a detailed OHNS triage system similar to the Manchester Triage System (MTS) [13] and the Australasian Triage Scale (ATS) [14], tailored to the specific context of OHNS ERs. We focused on the need for acute care in the ER rather than on process improvements or increased patient safety. The newly defined categories are summarized in Table 1.

Patients requiring immediate care due to potentially life-threatening symptoms, or the risk of permanent damage, were categorized as “Immediate,” corresponding to MTS and ATS categories 1 and 2. Symptoms without an immediate risk of severe damage but which could escalate into “Immediate” emergencies if left untreated were categorized as “Urgent” (MTS and ATS category 3). The category “Protracted” included patients with long-standing symptoms who did not require immediate or urgent care (MTS and ATS categories 4 and 5). Patients presenting with complaints that could have been managed by a general practitioner or those with non-urgent issues were grouped under “GP/non-urgent” (MTS and ATS category 5) (Table 1).

The diagnoses were grouped and ranked in percentiles. The collected data underwent demographic analyses and annual comparisons and were contextualized with government orders or events related to the COVID-19 situation in Germany (detailed in Supplement Table S1). Mean values with standard deviations (SD) were calculated. For statistical analysis, JMP version 14 was used. Statistical comparisons were performed using a one-sided t-test for independent samples (for normally distributed data) or a Mann–Whitney U test (for skewed data). A *p*-value of <0.05 was considered statistically significant.

## 3. Results

### 3.1. Characteristics of the Study Population

In the first two quarters (Q1, Q2) of 2018, 2019, and 2020, a total number of 11,277 patients were included in this retrospective analysis (Table 2). In 2018, a total of 1760 patients were treated in the ER (Q1: 916; Q2: 884) and 2113 in the EP (Q1: 1099; Q2: 1014). In 2019, the ER recorded a total of 1778 patient visits (Q1: 812; Q2: 966) and the EP 2342 (Q1: 1074; Q2: 1268). In 2020, a total of 1365 patients were treated in the ER (Q1: 807; Q2: 558), and 1879 patients were treated in the EP (Q1: 1159; Q2: 720) (Table 2).

Subsequently, a detailed analysis of the ER population during the period of the “first wave” between 1 March and 30 June of 2019 and 2020 was carried out. In 2019, 1229 patient visits to the ER were registered during the on-call hours (679 males, 55.2%; 550 females, 44.8%). The overall daily average was 10 patients (SD ± 6). In the same timeframe, in 2020, a total of 753 patients presented to the ER during on-call hours, with a daily average of 6 (SD ± 4) patients (435 males, 57.8%; 318 females, 42.2%). This resembles a significant reduction in overall cases (daily average 2019: 10.1, SD ± 6; daily average 2020: 6.2, SD ± 4, *p* < 0.0001).

**Figure 2:**
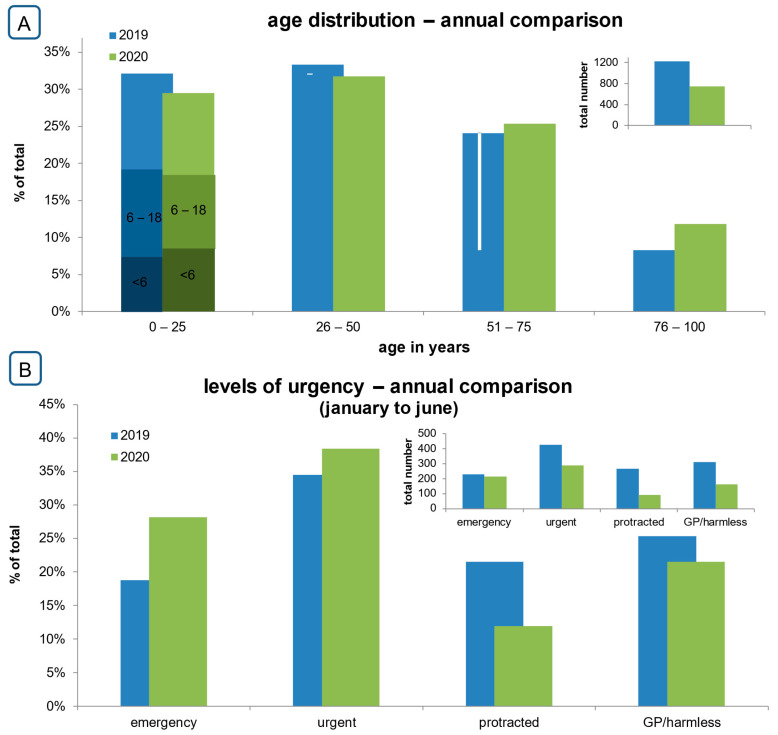
Annual comparison of patients presenting to the ER between March and June 2019 and 2020 depicted as a percentage of the total number for each year. The insets show absolute numbers. (**A**): Age distribution. Patients aged 0–25 years were additionally subdivided (age of >6, from 6 to 18, and 19 years or older). (**B**): Distribution according to the applied triage system.

In 2019, the average age was 38.6 (SD ± 22.9) years, which was significantly younger than that in 2020 (41.9 years; SD ± 24.6) (*p* = 0.0011) (Figure 2). In the annual comparison, the sex distribution did not differ significantly. Table 3 offers a detailed breakdown of the daily average in the different triage categories. 

### 3.2. The Number of Emergencies Declines after the Onset of the Pandemic

**Figure 3:**
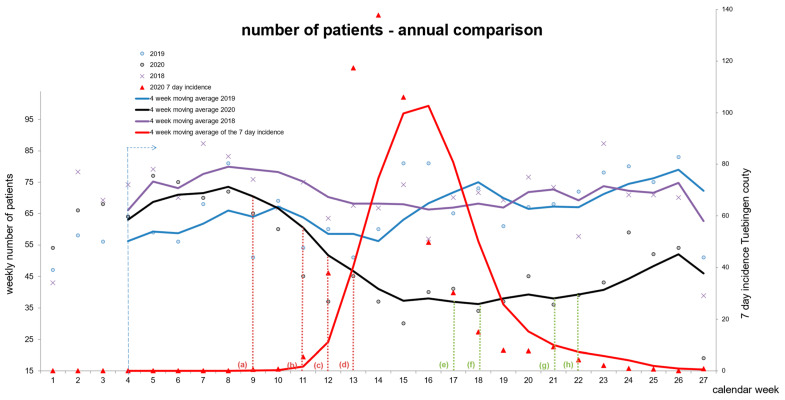
Annual comparison of numbers of patients during the first half of the years of 2018, 2019, and 2020. The dots represent the weekly amount in each calendar week. A moving average (4 weeks) was added and governmental restrictions and liberalizations regarding COVID-19 were marked in the graphic. The red triangles represent the weekly 7-day incidence of newly positive SARS-CoV-2 tests. The red line is the 4-week moving average for the 7-day incidence. (a) first infection in the state of Baden-Württemberg, (b) WHO declares global pandemic, (c) closure of places of worship/schools/universities/shops, (d) nationwide contact ban (only family or one additional person), (e) first liberalizations (reopening of shops), (f) reopening of museums/zoos/places of worship, increase in elective surgeries (g) 10 consecutive days with fewer than 1000 new infections, (h) appeal of the German Minister of Health requesting German citizens not to postpone necessary doctors’ appointments anymore (also see Table S1).

In 2020, we observed a distinctive U-shaped course of ER visits that followed the disease control restrictions and liberalization that were imposed by the federal and state governments (Figure 3). The daily average in ER cases during on-call hours was reciprocal to the number of daily new infections with SARS-CoV-2. This fluctuation in case numbers was not observed in 2018 and 2019. The daily average number of ER cases increased toward the summer of 2020, with a relevantly reduced SARS-CoV-2 incidence, but failed to reach pre-pandemic levels (Figure 3).

We analyzed the ratio between the ER and EP cases in quarters one and two (Q1, Q2). For the pre-pandemic years, this ratio was stable, and in 2020, the ratio decreased in Q2, indicating that the EP experienced a greater decrease in cases in Q2 than the ER (Table 2).

### 3.3. The Change in Patient Numbers Is Mainly Due to Protracted and Non-Urgent Cases

From March to July 2019, the largest proportion of patients required “Urgent” care (*n* = 424; 34.5%), followed by the category “GP/non-urgent” (*n* = 311; 25.3%), the group “Protracted” symptoms (*n* = 264; 21.5%), and “Immediate” emergencies (*n* = 230; 18.7%) (Figure 2B).

In 2020, the most prevalent group of patients was assigned to the “Urgent” category (38.4%; *n* = 298 cases), followed by the “Immediate” emergencies (*n* = 212; 28.2%), the “GP/non-urgent” group (*n* = 162; 21.5%), and patients with “Protracted” symptoms (*n* = 90; 12.0%) (Figure 2B).

The annual comparison revealed that the daily number of patients categorized as “Immediate” was similar in both years (daily average 2019 ± SD 1.9 ± 1.4, daily average 2020 ± SD 1.7 ± 1.5, *p* = 0.2371), but was significantly reduced in lower triage categories (“Urgent”, daily average 2019 ± SD 3.6 ± 2.2, daily average 2020 ± SD 2.8 ± 1.8, *p* < 0.0001; “Protracted”, daily average 2019 ± SD 3.0 ± 2.3, daily average 2020 ± SD 1.7 ± 0.9, *p* < 0.0001; “GP/non-urgent” daily average 2019 ± SD 3.1 ± 3.2, daily average 2020 ± SD 1.8 ± 1.2, *p* = 0.0011) (Table 3). Although the total number of “Immediate” emergencies was stable, the proportion of cases increased in 2020 (Figure 2B). A weekly analysis of the level of urgency is provided in the Supplement (Figure S1).

From March to July 2019, 231 patients were admitted (18.8%), and 72 underwent emergency surgery in direct relation to their diagnosis (5.8%). For 38 cases, postoperative complications were the reason for consultation (3.1%). In the same period, in 2020, 140 patients were admitted as inpatients (18.6%), and 52 underwent surgery based on their presentation to the ER (7.0%). Forty-three of the registered cases were due to postoperative complications (5.7%).

In summary, the absolute number of immediate emergencies in the pandemic remained stable, while the protracted and non-urgent cases decreased in absolute numbers and proportionally. The percentage of patients who were admitted to the inpatient ward was almost the same in both years, but the absolute number was reduced significantly (*p* = 0.0002).

### 3.4. Diagnoses with the Sharpest Decline Were All Inflammatory OHNS Diseases

Individual patient diagnoses were grouped into clusters. The most frequent diagnosis in both years was epistaxis, with a decline of only 3% from 2019 to 2020 (2019: *n* = 166; 2020 *n* = 161). The total number of each cluster and the proportional relation between 2019 and 2020 are shown in the table below (Table 4).

An examination of the changes from 2019 to 2020 revealed different patterns. Epistaxis, globus sensation/functional dysphagia, and foreign bodies showed little to no change (<10% decline). Abscesses, as well as cases of sudden hearing loss, slightly decreased. In 2020, the number of patients presenting with vertigo, tonsillar abscess, dyspnea, postoperative bleeding, traumatic injuries, or symptoms classified as “other” decreased by at least one quarter (10–50% decline). The number of skin infections, otitis externa, tonsillitis, sinusitis, and otitis media decreased most strongly. These five diagnoses all belong to the infectious diseases category, and numbers more than halved (>50% decline, Table 2).

The only diagnosis that increased from 2019 to 2020 was tumor bleeding. A closer examination showed that of the four cases in 2019, the time between initial cancer diagnosis and bleeding was a maximum of 4 months (average 2.75 months). One patient presented twice for hemorrhage; therefore, the four cases can be attributed to three patients. In two patients, primary curative therapy was planned or performed; in one patient, palliative therapy was planned, but not with checkpoint inhibitors.

During the same period in 2020, the intervals between the first cancer diagnosis and bleeding were more heterogeneous, ranging from 1 to 21 months (mean, 9 months). Three patients presented bleeding at least twice, so the 14 cases of tumor bleeding were attributable to 10 patients. Of these 10 patients, only two were on a curative treatment plan, whereas eight were considered palliative. In this group, five patients received systemic therapy with a checkpoint inhibitor, but only after the bleeding event.

## 4. Discussion

The COVID-19 pandemic had a significant impact on medical systems worldwide, especially on timely and appropriate emergency care. During the early days of the pandemic, governments were challenged to uphold optimal health care without having a template to use. This led to great uncertainty not only among policymakers, but also for the general public. Several studies have documented a reduction in emergency department cases during the first surge of COVID-19 cases [7,9,10,15]. Although they provide valuable insight into the development of cases, most studies are single-center reports and fail to differentiate cases based on urgency.

Addressing this gap, our study features a multi-center analysis of OHNS emergencies both at a tertiary care hospital and in an ENT emergency practice. We introduce a novel, specialized triage system tailored to otolaryngology. This offers the unique opportunity to systematically assess the urgency of ER presentation. Furthermore, by offering a longitudinal comparison across pre-pandemic and pandemic years (2018–2020), our study allows for a robust analysis of trends and impacts over time. This comprehensive approach not only enhances the representativeness and accuracy of our findings, but also contributes valuable insights into the specific changes in OHNS emergency care during the COVID-19 pandemic, filling a critical gap in the existing literature.

We not only examined the large number of 11,277 patients and the urgency of these cases, but also analyzed the individual diagnoses and compared these to the previous years. The number of ER consultations decreased rapidly, beginning with the onset of the acute threat posed by the first surge of COVID-19 cases in Germany (Figure 3). The biggest decline in cases was observed in the clusters of acute infectious diseases. Due to the very nature of these diagnoses, seasonal fluctuations are a major confounder [16]. A valid analysis of the variation in incidence can therefore not be based on a one-year observation. In addition to prior German [5,9] and international trials [17], which applied a 2020-specific approach, we assessed the level of urgency by introducing a new OHNS-specific triage system and included the years 2018 and 2019 in our analysis.

The overall number of patients presenting to the ER during the on-call hours decreased significantly in comparison to the pre-pandemic years (roughly 40% reduction). This effect was mainly driven by a sharp reduction in visits during the first surge of COVID-19 in Germany. The decrease was not uniformly distributed over the triage categories, but was driven by consultations allocated to the non-emergency categories (“Protracted”, “GP/non-urgent”). However, the absolute number of presentations in the “Immediate” category was stable. Concerning the percentage distribution among the triage categories, we observed a shift towards higher levels of urgency, with an increase in the “Immediate” (+9.4%) and “Urgent” (+3.9%) categories. The detailed data, including the diagnoses, represent the ER, a tertiary care provider. Although the analyzed consultation during on-call hours was mainly initiated based on self-referral, a confounding effect by expert referral cannot be ruled out. The ER represents a transregional tertiary care center that might receive referrals from secondary centers and also might be biased toward more urgent and more severe cases, whereas the EP is an urgent care outpatient service provided by private practitioners. Therefore, we analyzed the ratio between the ER and EP cases in quarters one and two (Q1, Q2). The inclusion of the EP as a second center corroborates the representativity. Consultations are based on self-referral in both centers. As shown in Table 2, the ratio between the ER and the EP was essentially stable.

With the first liberalizations of the disease control measurements, the frequency with which patients presented to the ER and the EP recovered, but failed to reach pre-pandemic levels. A similar effect was observed not only for OHNS emergencies, but also for general ER consultations [8,12].

The reasons for reduced ER consultations are diverse: disease control measurements intended to reduce the spread of SARS-CoV-2 are also effective for other (airborne) infections (e.g., common cold, flu). Widespread use of surgical masks is a proven intervention to reduce the spread of airborne, droplet, and smear infections, and proper hand hygiene reduces the overall risk of infections [18,19]. Our data document a significant reduction in infectious diseases (Table 4). This is consistent with other, more general data (e.g., absence of influenza A in 2020 and 2021) [20,21].

Today, we know that the lack of exposure to pathogens during the COVID-19 pandemic resulted in reduced immune stimulation, thereby weakening adaptive immunity to specific pathogens [22]. In addition, patients recovering from COVID-19 developed long-term SARS-CoV-2-specific immune and inflammatory responses, e.g., degranulating virus-specific CD3+8+ T cells with and without post-acute symptoms [23].

However, the OHNS infections in the present study were largely bacterial in origin. We observed an increase in complications of otitis media, such as mastoiditis and labyrinthitis, or sinugenic complications, such as orbital abscesses, at the University Hospital Tübingen in the post-COVID period. A systematic review of these data is planned.

Social distancing measures have led to changed leisure behavior. The stay-at-home policies can explain the significant reduction in traumatic injuries, e.g., nasal bone fractures or auricle injuries in 2020.

As with other global health crises, the COVID-19 pandemic has increased the level of experienced stress and other mental health issues [24]. Over 50% of the U.S. American population reported experiencing mental health effects (e.g., anxiety over being infected) [6,25]. This also applies to people without pre-existing mental health issues [24]. Therefore, and due to universal stay-at-home invocations, patients with minor complaints might have refrained from an ER visit.

In contrast, the prevalence of other diagnoses remained stable, particularly the number of epistaxis cases. This contrasts with other emergency diagnoses, such as acute myocardial infarction, which occurred significantly less during the COVID-19 pandemic [8,26,27]. Like epistaxis, the absolute number of patients diagnosed with functional problems such as globus sensation was stable. However, these cases were generally not categorized as emergencies and may be linked to psychological factors [28]. Over 90% of patients with globus sensation report an exacerbation of symptoms during times of emotional intensity [29].

The long-standing, pre-pandemic issue of emergency department overcrowding [1,2,30] and OHNS-specific emergency services [31] gained new acuity during the pandemic. This led to an emphasis on the need for reforms in the use of emergency services. The need for restricted physical contact led to the exploration of appropriate concepts, e.g., a strong emphasis on telemedicine [32,33] and the redirection and reorganization of the physical patient flow, such as through the establishment of rapid access (OHNS) clinics [31].

The adjustment of patients’ evaluation of their symptoms and the fear of becoming infected during social interactions in emergency departments may have reduced the cases to bare emergencies. “Inappropriateness” of emergency department consultations must be discussed with great care [30], but this study supports the hypothesis of inappropriate use in the pre-COVID-19 era, especially in OHNS [31]. Our analysis brings a new perspective to the longstanding issue of emergency department overcrowding. The significant reduction in non-urgent cases during the pandemic suggests that many pre-pandemic emergency department visits may have been inappropriate. This highlights the need for public education on appropriate emergency department use and the implementation of policies such as e-referrals and rapid access clinics to streamline patient flow and reduce the burden on emergency services. The first experiences with telemedical approaches have been shown to be effective in reducing visits [32,33]. Other concepts aim to strengthen the role of primary care providers in offering e-referrals that can lead to direct admissions and, therefore, reduce the need for emergency department visits [34] and the establishment of rapid access (OHNS) clinics [31]. Eventually, 9 months after the official end of the pandemic in April 2023, the German Ministry of Health presented key points to reform emergency care in January 2024 (https://www.bundesgesundheitsministerium.de/fileadmin/Dateien/3_Downloads/N/Notfallversorgung/Eckpunkte_Notfallreform_16.01.2024.pdf) (accessed on 22 May 2024), including the nationwide coordination of ERs, improved digital networking, and the increased integration of telemedicine.

The pandemic’s impact on healthcare-seeking behavior, particularly the delay in seeking medical consultation, has long-term implications. The increase in complications from untreated conditions and the delay in diagnosing serious illnesses like head and neck malignancies underscore the need for strategies to mitigate these effects in future health crises. One possible adverse effect of delaying medical consultation with new complaints is documented in the delayed diagnosis of head and neck malignancies during the COVID-19 pandemic [35]. The significant increase in OHNS carcinoma hemorrhages in 2020 in this study and in the interval between initial cancer diagnoses and bleeding events was only driven by an increase in palliative cases. This could be due to the delay in establishing the diagnoses because of the pandemic. Additionally, this shift in patient behavior may result in a sustained change in the demand for emergency services, necessitating ongoing adjustments in emergency care delivery.

Recent publications reported a significant increase in palliative therapies with immune checkpoint inhibitors in OHNS [36]. Although our data confirm this observation, the use of checkpoint inhibitors does not seem to lead to an increased bleeding risk.

This study is limited in its power, as we conducted a retrospective analysis. Therefore, we were not able to investigate from actual patients’ perspectives, e.g., using questionnaires or in-depth recording of demographic parameters (e.g., socioeconomic background). Although this was a multi-center trial with centers from different levels of emergency care (general EP and tertiary care ER), the centers cover the same medical specialty and the same geographic region. However, the combination of precise diagnoses and the newly established, OHNS-specific triage categories allows a critical analysis of ER use. This analysis places a new spotlight on the longstanding question of ER overcrowding. Similar effects have been observed in single-center trials in Germany [9] and other European centers, such as Italy [17] and Greece [15]. Here, almost all OHNS emergency categories, including epistaxis, experienced a significant decline. Interestingly, foreign body aspiration reached no statistical significance [15]. In our study, epistaxis cases were not significantly reduced. The same development holds for the foreign body category. This might be due to the longer observational period, the larger number of cases included in the study at hand, and the fact that while our center is the only OHNS emergency provider within a range of 50 km, in Athens, several centers provided OHNS emergency care. The strength of the present study is the combination of different centers, the evaluation of the precise diagnoses, and the individual categorization with an OHNS-specific triage system.

Our study’s insights also have significant implications for managing future global health crises. The observed trends and effective interventions, such as specialized triage systems and telemedicine, can inform international strategies for emergency response. A comparative analysis with similar studies from other countries could provide a broader perspective, facilitating international collaboration and information sharing to enhance global health preparedness.

## 5. Conclusions

The COVID-19 pandemic posed unprecedented challenges to global healthcare systems, particularly affecting the delivery of emergency care. Our multi-center study provides critical insights into the changes in otolaryngology, head and neck surgery (OHNS) emergencies during this pandemic period. The introduction of a specialized triage system allowed, for the first time, a nuanced assessment of OHNS emergency presentations, revealing a significant shift towards higher-urgency cases and a substantial reduction in non-urgent visits. Furthermore, the COVID-19 pandemic has irrevocably changed the landscape of emergency medicine. By understanding these changes and implementing effective strategies, we can enhance the delivery of emergency care and ensure preparedness for future health crises. Therefore, further research is needed to investigate the factors influencing patients’ decisions to seek or delay emergency care. This could provide valuable insights for public health strategies. In addition, the integration of telemedicine into routine emergency in OHNS-emergency care could help to optimize patient flow and reduce emergency department overcrowding.

## Figures and Tables

**Table 1:** Definition of triage categories.

OHNS Category	Definition	MTS/ATS Category (MTS Description)
Immediate	Conditions that could lead to permanent severe damage or death without instant treatment.	1 (Immediate) 2 (Very urgent)
Urgent	Conditions that impose no immediate mortal danger or acute risk for severe or permanent damage but could deteriorate into an “Immediate” emergency if medical care is not provided.	3 (Urgent)
Protracted	Symptoms or diseases that have been present for a prolonged period and do not require emergency or urgent care.	4 (Normal) 5 (Non-urgent)
GP/non-urgent	Conditions not requiring treatment by a medical specialist.	5 (Non-urgent)

**Table 2:** Number of patients presenting to the ER and EP.

	2018			2019			2020		
	ER	EP	Ratio EP/ER	ER	EP	Ratio EP/ER	ER	EP	Ratio EP/ER
Quarter 1	916	1099	1.20	812	1074	1.32	807	1159	1.44
Quarter 2	884	1014	1.15	966	1268	1.31	558	720	1.29
Difference	−3.5%	−7.7%		+18.9%	+18.1%		−30.8%	−37.8%	

The table lists the absolute number of consultations in the ER and EP, respectively. The EP/ER ratio was calculated to better appreciate potentially diverging trends in consultations. The last row shows the difference in consultations from the first to the second quarter for each year.

**Table 3:** Annual comparison of the daily patient numbers.

	2019	2020	Change in %	*p*
Immediate	1.9 (±1.4)	1.7 (±1.5)	−11%	*p* = 0.2371
Urgent	3.6 (±2.2)	2.8 (±1.8)	−22%	*p* < 0.0001
Protracted	3.0 (±2.3)	1.7 (±0.9)	−43%	*p* < 0.0001
GP/non-urgent	3.1 (±3.2)	1.8 (±1.2)	−42%	*p* = 0.0011

**Table 4:** Clusters of diagnoses in 2019 and 2020 ranked by change.

Percentiles	Cluster of Diagnoses	Amount 2019 (March to July)	Amount 2020 (March to July)	Difference 2019 -> 2020	Level of Significance
>0%	Tumor bleeding	4	24	+250%	0.0060 **
<10%	Epistaxis	166	161	−3.0%	0.6263
	Functional problem/globus sensation	31	30	−3.2%	0.9054
	Foreign body nose/ear	54	51	−5.6%	0.7790
10–50%	Abscess—other	14	12	−14.3%	0.8336
	Sudden sensorineural hearing loss	21	16	−23.8%	0.3805
	Vertigo	45	32	−28.9%	0.1072
	Abscess—tonsillar	31	21	−32.3%	0.2249
	Dyspnea	18	11	−38.9%	0.0851
	Postoperative bleeding	24	13	−45.8%	0.0688
	Other	370	197	−46.8%	<0.0001 ***
	Traumatic injury	107	55	−48.6%	0.0002 ***
50–100%	Skin infection	43	21	−51.2%	0.0060 **
	Otitis externa	73	35	−52.1%	0.008 ***
	Tonsillitis	81	36	−55.6%	0.0010 ***
	Sinusitis	39	16	−59.0%	0.0006 **
	Otitis media	108	32	−70.4%	<0.0001 ***

Clusters of diagnoses in 2019 and 2020 ranked by change in categories: >0% (increase of consultations), <0%, 10–50%, and 50–100% (decrease of consultations). Levels of significance: *** for *p* ≤ 0.001 and ** for *p* ≤ 0.01.

## Data Availability

All relevant data are included in the article. The original data cannot be shared due to privacy reasons.

## Supplementary Materials

The following supporting information can be downloaded at: https://www.mdpi.com/article/10.3390/diseases12080194/s1, Figure S1: Weekly analysis of levels of urgencies; Table S1: Timetable of Government disease control measures or events linked to COVID-19.
