# Supplementary material for: The COVID-19 Pandemic and Emergencies in Otolaryngology–Head and Neck Surgery: An Analysis of Patients Presenting to Emergency Rooms in South-West Germany: A Bi-Center Study
Source: Diseases. 2024 Aug 22;12(8):194. doi: 10.3390/diseases12080194 (PMC11354163; doi:10.3390/diseases12080194)
Supplement: Supplementary file 1 [file diseases-12-00194-s001.zip › diseases-3051589-supplementary.pdf]

Supplemental Material

Supplemental Table S1. Timetable of Government disease control measures or events linked to COVID-19.

Date	Government disease control measures or events linked to COVID-19
27 Feb 2020	a) Mandatory two-week quarantine for every contact person or travelers returning from risk areas
09 Mar 2020	b) First fatality due to COVID-19 in Germany
11 Mar 2020	c) World Health Organization (WHO) declares COVID-19 a pandemic, university lectures cancelled (anticipated reinstatement 19 April 2020)
17 Mar 2020	d) Closure of places of worship and educational institutions; prohibition and cancellation of events
20 Mar 2020	e) Closure of restaurants, shops and hairdressers (daily supplies excluded), reduction of allowed group size to three people, entry and transit ban for travelers from risk areas
23 Mar 2020	f) Nationwide contact ban (contacts limited to one person or family)
29 Mar 2020	g) Introduction of a catalogue of fines in case of violations of the contact ban
20 Apr 2020	h) First relaxation of regulations (partial reopening of educational institutions and shops up to 800 m^2)
27 Apr 2020	i) Mandatory usage of masks in public transportation and shops
30 Apr 2020	j) Further easing of restrictions regarding places of worship, playgrounds, museums and zoos
06 May 2020	k) Meetings of up to two households allowed in public spaces
13 May 2020	l) Reduction of border controls
19 May 2020	m) 10 consecutive days with fewer than 1000 new infections registered
30 May 2020	n) Appeal of the German Minister of Health requesting German citizens not to postpone necessary doctors' appointments anymore
09 Jun 2020	o) Testing of asymptomatic people allowed
16 Jun 2020	p) Introduction of the corona-warn-app (mobile phone application to alert users after contact to patients tested positive for COVID and to trace infection chains)

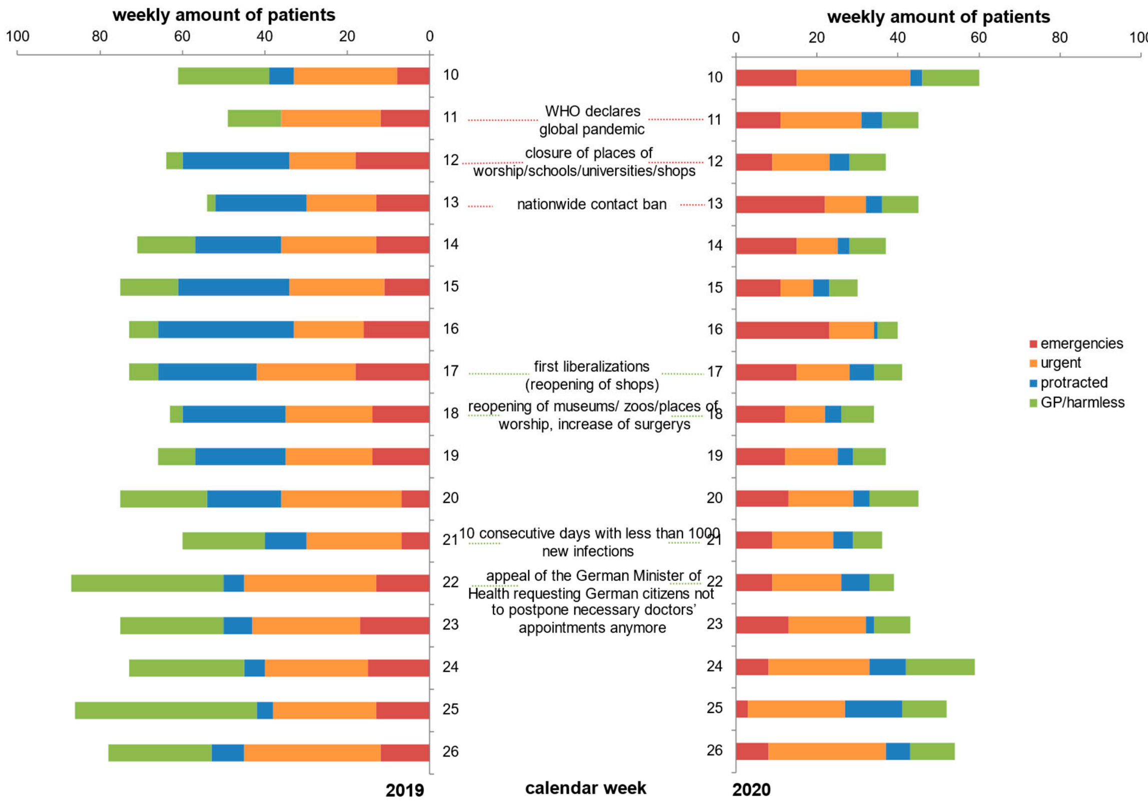

Supplemental Figure S1. Weekly analysis of levels of urgencies.
